# Gene function prediction using labeled and unlabeled data

**DOI:** 10.1186/1471-2105-9-57

**Published:** 2008-01-28

**Authors:** Xing-Ming Zhao, Yong Wang, Luonan Chen, Kazuyuki Aihara

**Affiliations:** 1 ERATO Aihara Complexity Modelling Project, JST, 4-6-1 Komaba, Meguro, Tokyo, Japan; 2 Intelligent Computing Lab, Hefei Institute of Intelligent Machines, Chinese Academy of Sciences, Hefei, Anhui, 230031, China; 3 Institute of Industrial Science, The University of Tokyo, Tokyo, Japan; 4 Department of Electrical Engineering and Electronics, Osaka Sangyo University, Osaka, Japan; 5 Institute of system biology, Shanghai University, Shanghai, China

## Abstract

**Background:**

In general, gene function prediction can be formalized as a classification problem based on machine learning technique. Usually, both labeled positive and negative samples are needed to train the classifier. For the problem of gene function prediction, however, the available information is only about positive samples. In other words, we know which genes have the function of interested, while it is generally unclear which genes do not have the function, i.e. the negative samples. If all the genes outside of the target functional family are seen as negative samples, the imbalanced problem will arise because there are only a relatively small number of genes annotated in each family. Furthermore, the classifier may be degraded by the false negatives in the heuristically generated negative samples.

**Results:**

In this paper, we present a new technique, namely Annotating Genes with Positive Samples (AGPS), for defining negative samples in gene function prediction. With the defined negative samples, it is straightforward to predict the functions of unknown genes. In addition, the AGPS algorithm is able to integrate various kinds of data sources to predict gene functions in a reliable and accurate manner. With the one-class and two-class Support Vector Machines as the core learning algorithm, the AGPS algorithm shows good performances for function prediction on yeast genes.

**Conclusion:**

We proposed a new method for defining negative samples in gene function prediction. Experimental results on yeast genes show that AGPS yields good performances on both training and test sets. In addition, the overlapping between prediction results and GO annotations on unknown genes also demonstrates the effectiveness of the proposed method.

## Background

One of the main goals in post-genomic era is to predict the biological functions of genes. Recently, with the rapid advance in high-throughput biotechnologies, such as yeast two-hybrid systems [1], protein complex [2,3] and microarray expression profiles [4], a large amount of biological data have been generated. These data are rich sources for deducing and understanding gene functions. For example, protein-protein interaction data are widely exploited for inferring functions of genes with the assumption that interacting proteins have the same or similar functions, i.e. "guilty by association" rule [5-10]. In addition, gene expression data have been widely used for gene function prediction, where genes with similar expression patterns are assumed to have similar functions [11]. In the literature, it has been shown that integration of different kinds of data sources can considerably improve prediction results [12-15]. With various kinds of high-throughput data, the machine learning techniques, especially Support Vector Machines (SVMs), have been used for predicting gene functions and shown promising results [16,17].

Despite the good performance of the machine learning techniques, there are some limitations with existing methods, where gene function prediction is formalized as a classification problem. Generally, to construct a classifier for gene function prediction, one needs a number of labeled training samples. In this case, it is relatively easy to find positive samples (i.e. genes annotated with the function of interested) that have been annotated by human experts. However, it is hard to find the representative negative samples because the available information in the annotation databases, such as Gene Ontology (GO) [18] and the Munich Information Center for Protein Sequences (MIPS) [19], is only about positive samples, i.e. we know which gene belongs to which functional class but we are not sure which gene does not belong to the class. Hence, the available information to us is a set of genes that have the target function, whereas it is unclear whether the other genes have the function or not. Furthermore, since one gene may be annotated by more than one function, it is inappropriate to use all the other genes outside of the target functional class as negative samples (e.g. some of them may actually have such a function). In addition, the imbalanced problem will arise if all the genes outside of the target functional family are seen as negative samples because usually there are only a relatively small number of genes annotated with the function, while the number of negative samples may be hundreds even thousands times the one of positive samples. Therefore, the classifier may be degraded by the false negative samples or imbalanced data [20].

In this paper, a new technique, namely Annotating Genes with Positive Samples (AGPS), is presented for defining negative samples in gene function prediction. In particular, a functional linkage graph is constructed to integrate heterogeneous information sources and the singular value decomposition (SVD) technique is employed to reduce dimensionality and remove noise from the data. Then, the AGPS algorithm is presented to define negative samples and predict functions of unknown genes. In this work, genes annotated with the target function are denoted as labeled data, while those annotated with other functions instead of the target annotation are denoted as unlabeled data. The unlabeled data are defined here because we assume that the genes without target annotation may have the function even though they are currently not annotated with the function. The goal of the AGPS algorithm is to automatically generate negative samples from unlabeled data in the learning procedure. Unlike the conventional single-class learning algorithm that is trained only on positive samples, e.g. one-class SVMs [21], the AGPS algorithm defines negative samples from unlabeled data automatically in the learning procedure, and therefore is expected to have a superior performance. With SVMs as the core learning algorithm, the AGPS algorithm can predict the functions of unknown genes effectively. Recently, the basic idea has also been employed by other researchers [22-26], where promising performances have been demonstrated. The major difference between AGPS and the existing algorithms [22-26] is that instead of recognizing the positive samples from the unlabeled data directly, the AGPS algorithm aims to define the representative negative samples from unlabeled data. With the negative samples available, it is straightforward to predict the functions of unknown genes by utilizing both the positive samples and the defined negative samples. In addition, to exploit all available information, the AGPS algorithm can also integrate various kinds of data sources from high-throughput technologies so as to predict gene functions in a reliable manner.

To demonstrate the effectiveness, the proposed method is applied to predict functions of *S. cerevisiae *genes in the following procedures. Firstly, the data from protein interaction network, gene expression profiles and protein complex data are integrated into a functional linkage graph. Secondly, SVD is used to reduce the dimensionality and remove noise by extracting the dominant structure of the functional linkage graph. Finally, the AGPS algorithm is employed to predict the gene functions based on the refined data.

## Results and Discussions

### Data sources and processing

In this study, three kinds of data were integrated into a functional linkage graph for function prediction of *S. cerevisiae *genes. The three data sources include protein-protein interaction, gene expression profiles and protein complex data.

#### Functional annotation

The functional annotation data of *S. cerevisiae *genes used here were obtained from the FunCat 2.0 [27] functional classification scheme in 2006, which can be downloaded from the Comprehensive Yeast Genome Database (CYGD) of MIPS [19]. The annotation data in FunCat are organized as a hierarchical and tree like structure with up to six levels of increasing specificity. In total, the FunCat includes 1307 functional categories. A protein annotated by one function in the functional tree is also annotated by all the parents of the functional node. In this work, 13 general functional classes were selected, and consequently 4049 genes have been annotated in total. Table 2 shows the selected functional classes and the corresponding number of genes.

**Table 2:** The functional categories and genes used in this paper

Functional categories	Number of genes
01 metabolism	967
02 energy	241
10 cell cycle and DNA processing	727
11 transcription	829
12 protein synthesis	364
14 protein fate	680
20 cellular transport	726
30 cellular communication	86
32 cell rescue, defense and virulence	307
34 interaction with the environment	332
40 cell fate	201
42 biogenesis of cellular components	471
43 cell type differentiation	354

#### Protein interaction data

The protein interaction data used here were obtained from the BioGRID database [28]. The 2.0.20 version of BioGRID for yeast was used in this work. The dataset contains 82,633 pairs of interactions among 5,299 yeast genes, of which 4049 genes are annotated by the 13 functional classes. The protein-protein interaction can be represented as a network, where the vertices are genes and the edges are interactions among genes.

#### Gene expression profiles

The gene expression dataset used in this work was downloaded from the Stanford Gene expression Database (SMD), which contains the results from [29-33]. The missing values in the gene expression profiles were estimated by the *KNNimpute *algorithm [34], where *k *was set at 15. The dataset contains 6012 common genes, where 5,132 genes are among the 5,299 genes in the protein interaction dataset. Consequently, the dataset used in this work contains 5,132 genes with 278 real value features for gene expression data.

#### Protein complexes

The protein complex data were obtained from the MIPS database in 2006, including the data from [2] and [3]. The protein complex data were used here because genes occurring in the same complex are assumed to have the same or similar functions. Although we cannot infer direct interaction relationship among genes from protein complex data, the genes occurring in the same complex are generally considered to have functional correlations. Hence, we assigned functional relationships to genes occurring in the same complex, where an edge was constructed for a pair of genes occurring in the same complex. Finally, 62,042 functional edges were assigned to our dataset.

#### Data preprocessing

The relationship between any pair of genes in the protein interaction and complex datasets generated above was denoted as binary relationship here, because the relationship is only expressed by "yes" or "no", i.e. "yes" if two genes interact or occur in the same complex (where an edge was constructed), otherwise "no". The network with the binary relationship was denoted as a binary network. Unlike existing methods that utilize the binary network for gene function prediction, we used the binary network to estimate the functional similarities among genes. Specifically, to evaluate the functional similarity between a pair of genes, the Czekanowski-Dice distance (CD-distance) [35] was employed in this work. The CD-distance between genes *g*1 and *g*2 is defined as:

(1) $$D(g1,g2)=\frac{\left|N_{g1}\Delta N_{g2}\right|}{\left|N_{g1}\cup N_{g2}\right|+\left|N_{g1}\cap N_{g2}\right|}$$

where *N*_*g *_means the set containing gene *g *and its interacting partners, *N*_*g*1 _∪ *N*_*g*2 _means the union of *N*_*g*1 _and *N*_*g*2_, *N*_*g*1 _∩ *N*_*g*2 _means the intersection of *N*_*g*1 _and *N*_*g*2_, and *N*_*g*1_Δ*N*_*g*2 _means the symmetric difference between two datasets *N*_*g*1 _and *N*_*g*2_. The network with edges accompanied by functional similarity was denoted as the functional network in this paper.

For the protein interaction network, it is straightforward to apply the CD-distance. After that, the functional similarity between any pair of genes was represented as a real value between 0 and 1. The smaller the value, the more likely the pair of genes have the similar function. The functional network obtained from protein interaction data was denoted as *G*1.

For the gene expression profiles, the Pearson correlation coefficients were first calculated, and a binary network was then constructed for the dataset, where an edge was added if the absolute value of the correlation coefficient between corresponding pair of genes is larger than 0.7. In such a way, one binary network can be generated. Subsequently, the CD-distance was applied to the binary network just as described above. The obtained functional network from gene expression data was denoted as *G*2.

For the protein complex data, the CD-distance was applied directly to the binary network, and the resulted functional network was denoted as *G*3. Finally, we got three functional networks for yeast, and the functional similarity between any pair of genes in all of the three networks was measured by CD-distance. Furthermore, the three functional networks obtained in this way were merged into one integrated functional network *G *= *αG*1 + *βG*2 + *γG*3, where *G *is an *M *× *M *matrix and *M *is the number of genes. A simple rule *α *: *β *: *γ *= 1 : 1 : 1 was employed in this work. The idea behind the simple rule is that all the three data sources contain functional relationships among genes, and integrating them into one functional network can complement each other. The resulted functional network was denoted as the functional linkage graph in this paper.

### Results of 10-fold cross-validation on training data

With the functional linkage graph generated above, the AGPS algorithm was applied to predict functions of *S. cerevisiae *genes. Here, genes annotated with the target function **F **were regarded as positive samples or labeled samples, while those annotated with other functions instead of **F **were seen as unlabeled samples. Our aim is to define the negative samples and then predict the potential genes that may be annotated with **F **from the unknown genes. In the following experiments, gene function prediction was formalized as a multi-class classification problem, which was then reduced to a set of binary classification problems ("one vs the other" here). All the experiments were conducted by utilizing the LIBSVM [36].

First, we evaluated the proposed method on the training set [see Additional file 1]. In this paper, the training set consists of genes annotated in the MIPS annotation of March 2004, where 3663 genes have been annotated by the selected 13 functional classes (i.e. Table 2). To see the effectiveness of the defined negative samples on function prediction, AGPS was compared against four other methods including conventional two-class SVMs, one-class SVMs, PSoL [23], and kernel integration that is a simplified version of the one described in [16]. Furthermore, dimensionality reduction was performed to reduce computation cost and complexity. The SVD technique was employed to reduce the dimensionality and remove noise in this work, where 10-fold cross-validation was adopted to determine the number of components that should be kept in dimensionality reduction. For fair comparison, dimensionality reduction was performed for all the methods but kernel integration to find the informative components. No dimensionality reduction was performed for kernel integration method due to its specific data structure. The Radial Basis Function (RBF) kernel was employed for all the methods used in this work. The number of features selected for different methods can be found in Table 3.

**Table 3:** The number of features used for each class in the paper

Functional categories	Number of features				
	
	AGPS	PSoL	one-class SVMs	two-class SVMs	two-class SVMs_balanced
01 metabolism	295	110	10	10	10
02 energy	115	210	10	60	145
10 cell cycle and DNA processing	175	160	10	10	10
11 transcription	280	210	10	260	10
12 protein synthesis	25	160	260	260	10
14 protein fate	160	160	10	10	295
20 cellular transport	190	260	10	60	10
30 cellular communication	70	160	10	110	70
32 cell rescue, defense and virulence	295	160	10	110	250
34 interaction with the environment	85	210	10	10	295
40 cell fate	55	260	10	10	100
42 biogenesis of cellular components	25	260	10	10	190
43 cell type differentiation	40	210	10	10	40

For AGPS, the 10-fold cross-validation was employed to find the optimal parameters for kernel function, i.e. the positive set was randomly divided into 10 groups, where 9 of 10 subsets from the positive set were used as the positive training set, while the rest one was seen as a validation set. Both the validation genes and those outside of the target functional family were seen as unlabeled data. The learning procedure of AGPS in each trial of 10-fold cross-validation can be found in Table 1. In each trial of 10-fold cross-validation, the best classifier and the corresponding negative set were returned. Consequently, the negative samples occurring most often in the returned negative sets were taken as the representative negative samples, and the size of the final negative samples was controlled to nearly equal to that of the positive set. For example, after the 10-fold cross-validation, 10 negative sets were returned and the samples were ranked according to the times that they occurred in the 10 negative sets. Finally, the samples with the highest frequency were selected as the representative negative set. By selecting the negative samples in this way, the false negatives can be reduced. The final 10-fold cross-validation results were obtained based on the positive set and the defined negative set with the parameters determined above, which works in the same way as the conventional two-class SVMs does.

**Table 1:** Annotating Genes with Positive Samples (AGPS)

**Input:**
- positive training data **P1**
- validation set **P2**
- unlabeled data **Ku**
- unknown gene **Ug**
**Output:**
- Prediction results
**Stage 1: **Learning
**U **= **Ku **+ **P2**;
Stage 1.1: Initial negative set generation
- Construct classifier *f*_1 _based on **P1 **and **U **with one-class SVMs;
- Classify **U **using *f*_1_. The predicted negative set **N**_**1 **_is used as the initial negative training set in Stage 1.2;
- **U **= **U **- **N**_**1**_.
Stage 1.2: Negative set expansion
- Classifier set *FC *= [ ], negative set *NS *= [ ], *i *= 1.
- repeat
- *i *= *i *+ 1;
- Construct classifier *f*_*i *_based on **P1 **and **N**_**1 **_with two-class SVMs;
- *FC*(*i *- 1) = *f*_*i*_, *NS*(*i *- 1) = **N1**;
- Classify **U **by *f*_*i*_, **N**_**2 **_is the predicted negative set, where |**N**_**2**_| ≤ *k*|**P1**|;
- **N**_**1 **_= [**N**_**2**_; **N**_**SV**_], where **N**_**SV **_is the negative SVs of *f*_*i *_in the previous step;
- **U **= **U **- **N2**.
- until |**U**| <*k*|**P1**|
Stage 1.3: Classifier and negative set selection
- Classify **U **with classifiers from *FC*, and select the classifier *FC*(*i*) with the best prediction accuracy;
- Return negative set **TN **← *NS*(*i*).
**Stage 2: **classification
Classify **Ug **with **P **and **TN**, where **P **= **P1 **+ **P2**.

For PSoL, the 10-fold cross-validation was employed to find the optimal parameters for kernel function. In PSoL, the unlabeled data were defined to include those genes outside of the target functional class, unknown genes and validation genes. The learning of PSoL was implemented in the similar way as AGPS except that PSoL has only the learning stage in Table 1 and returns possible positive samples at the end of learning and does not select classifier. After the cross-validation, the best parameters were determined for PSoL and the best results were recorded. The details of PSoL can be found in [23].

For one-class SVMs, the genes outside of the target functional class were seen as negative samples and the classifier was trained only on the positive training set. The 10-fold cross-validation was utilized to find the optimal parameters for kernel functions, where 9/10 of the positive set was used as the positive training set and the rest was seen as positive validation set. Furthermore, one randomly-selected negative subset was used as the negative validation set, where the randomly-selected negative subset has nearly the same size as the positive validation set (i.e. 1/10 of the positive set). With the cross-validation, the best parameters for one-class SVMs were determined and the best results were recorded.

For two-class SVMs, the negative samples consist of genes outside of the target functional class. The 10-fold cross-validation was utilized to find the parameters with which they can best separate the positive samples from the negative samples. Furthermore, a balanced training set was generated for the two-class SVMs, where the negative samples with the same size as the positive samples were randomly selected from the genes outside of the functional class, and this technique has been used to define negative samples in the literature [37].

For the kernel integration method, the diffusion kernel was applied to binary networks generated by protein-protein interaction and complexes while the RBF kernel was applied to gene expression profiles. The parameters of the kernels were determined by 10-fold cross-validation. Instead of utilizing the semi-definite programming to find the optimal wight coefficients as descried in [16], the kernel matrices obtained were normalized and simply added together to form a new kernel. Furthermore, a balanced training set was generated, where the negative samples with the same size as the positive samples were randomly selected from the genes outside of the functional class.

Note that we compared different methods here to investigate the influence of different negative samples on gene function prediction, in particular to see the effectiveness of the defined negative samples by the proposed method on gene function prediction. In other words, the purpose of the comparison is not to demonstrate the superiority of the proposed method over existing methods but to show the effectiveness of the AGPS on defining negative samples for gene function prediction.

The results of 10-fold cross-validation by the five methods were shown in Table 4, which includes the results by two-class SVMs and kernel integration on balanced data. It can be seen from Table 4 that the AGPS algorithm performs comparably well with other methods due to the defined negative samples. Furthermore, all the methods utilizing the negative samples outperform the one-class SVMs that was trained only on positive samples. The poor performance of the one-class SVMs is due to the relatively fewer positive training samples. For the two-class SVMs and kernel integration, it can be clearly seen that with balanced data, the performance of both classifiers can be considerably improved. In other words, the imbalanced data can indeed degrade the performance of the classifiers. Furthermore, the results on imbalanced and balanced data demonstrate the importance of selecting negative samples in gene function prediction. Compared with PSoL, the AGPS algorithm can get a higher recall, i.e. it can recognize more positive samples hidden in the unknown data, because it defines better representative negative samples in the learning procedure.

**Table 4:** The results of 10-fold cross-validation by the five methods averaged over 13 classes

Methods	*precision*(%)	*recall*(%)	*F*1(%)
AGPS	68	61	61
PSoL	68	37	47
two-class SVMs	45	24	33
two-class SVMs_balanced	61	70	69
one-class SVMs	50	21	31
kernel integration	58	28	37
kernel integration_balanced	64	47	52

### Results on old data

Since March 2004, 386 previous unknown yeast genes have been annotated by the selected 13 functional classes (in 2006). Hence, the 386 genes were not involved in the training procedure in the previous section [see Additional file 2]. To validate AGPS and other methods, these 386 genes were regarded as test set and used to validate the models trained in the previous section. For the test data, the AGPS algorithm works as a conventional two-class SVMs here with parameters and negative set defined above. For PSoL, the unlabeled data were defined to include unknown genes and those genes outside of the target functional class. Note that the test data were included in unknown genes in PSoL. With the best parameters determined in the training procedure and all positive samples, PSoL was applied to find out putative positive samples from unknown genes. For the other three methods, the classifiers trained above were just utilized to predict the functions of unknown genes. Specifically, the two-class SVMs and kernel integration trained on imbalanced and balanced data were separately applied to predict gene functions. Furthermore, the *ROC *score, i.e. the area under the ROC curve, was also utilized to evaluate the overall performance of the classifiers. The *ROC *score was not used in the previous section because there are not negative samples available for single class methods. The results are shown in Table 5.

**Table 5:** The prediction results by the five methods averaged over 13 classes

Methods	*precision *(%)	*recall *(%)	*F*1 (%)	*ROC *score	coverage ^*a*^
AGPS	15	66	22	0.61	13 (13)
PSoL	20	18	19	0.55	12 (13)
two-class SVMs	28	10	16	0.53	11 (13)
two-class SVMs_balanced	18	36	29	0.57	10(13)
one-class SVMs	10	42	15	0.53	13 (13)
kernel integration	39	16	23	0.56	11(13)
kernel integration_balanced	11	32	24	0.59	6(13)

^*a *^The digit in the parenthesis is the true number of functional classes whereas the number outside is the number of classes that can be predicted by the corresponding method.

It can be seen from Table 5 that the AGPS algorithm outperforms all the other methods with respect to the overall performance, i.e. *ROC *scores. The poor performance of one-class SVMs is caused by the relatively small number of positive training samples, which result in underfitting. The PSoL algorithm performs well because it defines the negative samples like the AGPS algorithm. However, the selected negative samples and predicted positive samples by PSoL may not be true. On the other hand, the AGPS algorithm defines the representative negative samples that can best recognize the positive samples from the unlabeled data instead of predicting positive samples from the unlabeled data directly, which is also the reason why the AGPS algorithm can achieve a much higher recall rate. Working in this way, the AGPS algorithm is able to reduce false negatives. The results on balanced and imbalanced data demonstrate again that the two-class SVMs and kernel integration can be degraded by the imbalance problem. On the other hand, the difference between results on balanced and imbalanced data also shows the importance of selecting negative samples in gene function prediction. Although the two-class SVMs and kernel integration have higher *F*1 scores, they have lower recall rates compared against AGPS. However, a higher recall is much important because the biologists are mainly interested in which genes have the target function instead of which genes not. Although the imbalanced problem is avoided, both two-class SVMs and kernel integration trained on balanced data do not perform as well as the AGPS algorithm because the randomly selected negative training samples cannot capture the true distribution of negative samples very well.

**Figure 1:**
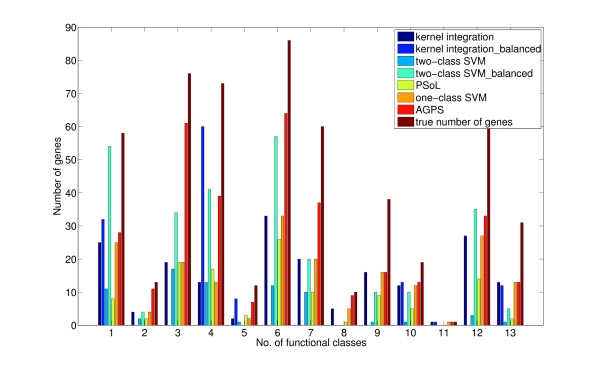
**The number of genes predicted correctly for the 13 functional classes**. The prediction results obtained by the five methods: AGPS, PSoL, two-class SVMs, one-class SVMs and kernel integration methods, where two-class SVMs_balanced means the results by two-class SVMs trained on balanced data and the same for kernel integration. The height of the bar in the figure means the number of genes that the five methods can recover correctly from unlabeled genes for each functional class, respectively.

To see the ability of the five methods to recover positive genes from unknown data, we compared the number of genes that the five methods can predict correctly from unknown genes on each functional class, where the results by both two-class SVMs and kernel integration trained on balanced data were also included. Figure 1 shows the results obtained by the five methods, where two-class SVMs_balanced means the results by two-class SVMs trained on balanced data and the same for kernel integration. It can be easily observed from Figure 1 that the AGPS algorithm can recover most of the unknown genes for nearly each functional class.

**Figure 2:**
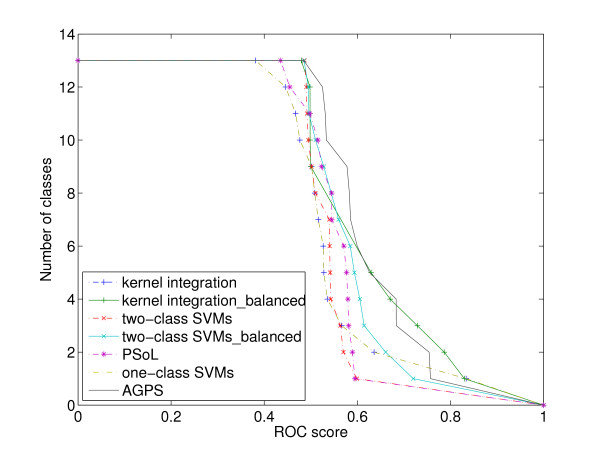
**Comparison of the five methods class by class**. Comparison of the performance among the five methods, where two-class SVMs_balanced means the results by two-class SVMs trained on balanced data and the same for kernel integration. The number of classes versus one *ROC *score threshold is countered, and a higher curve means a better result.

Furthermore, we compared our method against other methods class by class. The number of classes versus one *ROC *score threshold was countered, and a higher curve means a better result. Figure 2 shows the results by the five methods, where two-class SVMs_balanced means the results by two-class SVMs trained on balanced data and the same for kernel integration. It can be seen from the results that the proposed method outperforms all the other methods in this case, which confirms the effectiveness of the proposed method.

**Figure 3:**
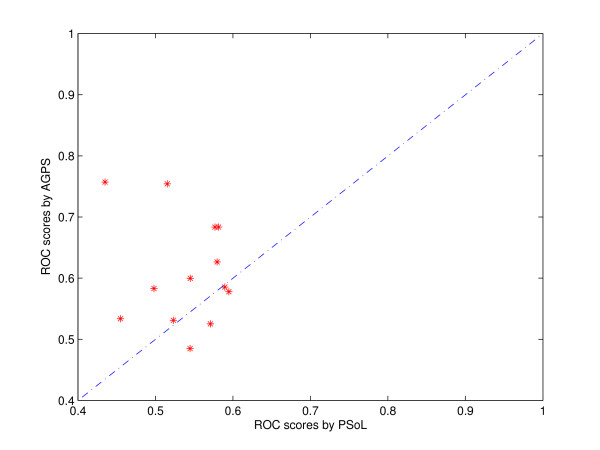
**Comparison of AGPS and PSoL class by class**. Comparison of the performance between the two single-class methods, i.e. AGPS and PSoL, class by class. The *ROC *scores obtained by the two methods for each functional class are compared.

In addition, we compared the performance of the best two single-class methods, i.e. AGPS and PSoL, class by class. Figure 3 shows the comparison of the *ROC *scores by the two methods on each functional class. It can be seen from Figure 3 that the AGPS algorithm outperforms PSoL on nearly each class, which also verifies the effectiveness of the proposed AGPS algorithm.

### Predicting functions of unknown genes

For the MIPS annotation data in 2006, there are 802 genes marked as not annotated in our dataset. With all the annotated genes in each functional class as the positive training set and the defined negative genes, the AGPS algorithm was applied to predict the functions of the 802 unknown genes. Although these genes are not annotated in the MIPS database, some of them may be annotated in the GO database with different functional terms. The GO annotations in 2006 were downloaded from GO database, and the GO terms assigned to the unknown genes were mapped to the MIPS annotation terms using the "go2mips" mapping table from GO. Finally, 457 genes annotated by GO can be mapped to MIPS, among which 21 genes have been annotated by 8 of the selected 13 functional classes [see Additional file 3].

To validate the predictions, the predicted results of AGPS were compared against the GO annotations for the unknown genes. Table 6 shows the results of the predicted terms versus GO terms, where only the predicted annotations that match GO terms with the same MIPS annotation are shown. It can be seen from Table 6 that the functions of nearly 71% (15/21) of the annotated genes from GO can be successfully recovered by AGPS, which confirms again the efficiency and effectiveness of the proposed algorithm.

**Table 6:** Predicted annotations by AGPS algorithm versus annotations from GO

MIPS functional categories	Gene ontology	genes annotated by GO	genes predicted by AGPS that match GO annotation
01 metabolism	GO:0008152	YEL044W YHL029C YGL185C YMR010W	YHL029C YGL185C YMR010W
10 cell cycle and dna processing	GO:0007067 GO:0006260 GO:0006281	YDR106W YGL168W YER038C	YDR168W YER038C
11 transcription	GO:0006364 GO:0006396	YLR196W YLR204W	YLR196W
12 protein synthesis	GO:0043037	YFR032C YLR287C	YFR032C YLR287C
14 protein fate	GO:0006457	YNL310C	YNL310C
20 cellular transport	GO:0006888	YDL099W	YDL099W
32 cell rescue, defense and virulence	GO:0006974 GO:0006950 GO:0006979	YOL063C YMR251W YDR346C	YOL063C YMR251W YDR346C
42 biogenesis of cellular components	GO:0019898 GO:0007005 GO:0007047	YPL005W YDR339C YNL149C YKR100C YNL310C YOR060C	YKR100C YOR060C

The predicted terms versus GO terms, where only the predicted annotations that match GO terms with the corresponding MIPS annotations are shown.

## Conclusion

Annotating genes with biological functions is one of the main goals in post-genomic era. An alternative way to this problem is to formalize gene function prediction as a classification problem. In this paper, we proposed a new algorithm, namely AGPS, to define negative samples in gene function prediction. The AGPS algorithm is different from existing methods, which have inappropriate assumptions about those genes that have no target annotation. Specifically, we do not simply regard those genes without target annotation as negative samples because one gene generally has multiple functions and it may indeed have the function even though it is not annotated with the target function currently. Unlike conventional binary classifier which needs both definite positive and negative samples, the new technique presented in this paper annotates genes by requiring only positive samples which are available in the public database. With explicit positive samples, the AGPS algorithm can define representative negative samples automatically in the learning procedure. Utilizing the defined negative samples, the AGPS algorithm performs comparably well with the existing methods in terms of prediction accuracy. In addition, the proposed method is able to integrate various kinds of data sources to infer gene functions in a reliable and accurate manner. In particular, by integrating the heterogeneous data from protein interaction, microarray profiles and protein complexes into a functional linkage graph, the AGPS algorithm was applied to predict functions of yeast genes in this paper. Furthermore, the SVD technique was utilized to reduce the dimensionality and remove noise, thereby significantly improving computational efficiency. Experiment results on yeast genes show that the prediction results on novel genes considerably overlap with GO, which confirms the effectiveness and the efficiency of proposed method.

What worth mentioning is that other kinds of data, e.g. protein subcellular localization and protein domains, can also be easily integrated for gene function prediction in our method. Furthermore, the AGPS algorithm can be expected to be applied to other fields in bioinformatics except gene function prediction. In this paper, we only applied the AGPS algorithm to the general functional classes. In the future, we will apply AGPS to more specific functional classes. In addition, the functional classes are considered independently here. However, generally there are correlations among these biological functions. If one gene has function *f*1, it is possible that the gene also has function *f*2 because function *f*1 correlates with function *f*2. Therefore, the performance will be improved if the correlation among genes is taken into account. The correlation among functions will be considered in the future work.

## Methods

**Figure 4:**
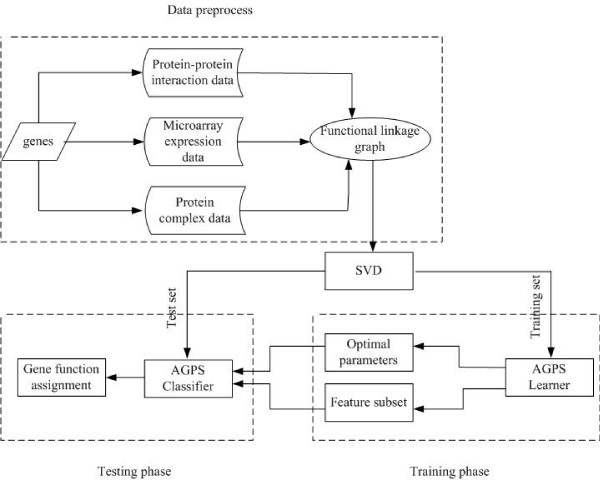
**Schematic flow chart of the proposed method**. Schematic flow chart of the proposed method. First, the protein interaction data, gene expression profiles and protein complex data for yeast genes are integrated into one functional linkage graph; Then, the SVD technique is utilized to project the gene vectors into low-dimensional feature space by uncovering the dominant structure of the functional linkage graph; Finally, the AGPS algorithm is utilized to predict the functions of genes.

In this section, we present a new method for defining negative samples in gene function prediction. Figure 4 gives an overview of the proposed method: (1) First, the protein interaction data, gene expression profiles and protein complex data for yeast genes are integrated into one functional linkage graph; (2) Then, the SVD technique is utilized to project the gene vectors into low-dimensional feature space by uncovering the dominant structure of the functional linkage graph; (3) Finally, the AGPS algorithm is utilized to define negative samples and to predict the functions of genes. The detailed procedure of the proposed method is given in the following subsections.

### Singular Value Decomposition

With the functional linkage graph generated previously, a total of 5132 features are generated for each gene. Compared to the number of features, the number of samples in each class lies in the range of (76, 909). Generally, too many features may significantly increase computation cost or make the classification problem much hard. Hence, it is necessary to extract the informative features and discard the effect of the noise. However, without any information on negative samples, the common feature selection methods cannot be used here. In this paper, the Singular Value Decomposition (SVD) technique is employed to uncover the dominant structure of the functional linkage graph. Recently, the SVD method has also been successfully applied to find functional clusters in large network [38,39], where it is shown that the SVD technique is effective to find the dominant structure of the network.

In SVD, given a matrix **A **of size *M *× *N*, **A **can be decomposed into three matrices:

(2) **A **= **SΣV**^*T*^

where **S **is the left singular matrix of size *M *× *K *(*K *is the rank of matrix **A**), **V **is the right singular matrix of size *N *× *K*, and **Σ **is the diagonal matrix of size *K *× *K *with non-negative eigenvalues *λ*_1 _> *λ*_2 _>,...,> *λ*_*K *_= 0. In this paper, *A *= **G **(i.e. the adjacent matrix of the functional linkage graph), and *M *= *N*.

After applying SVD to the matrix **G**, one can express **G **as follows:

(3) $$G=\sum_{r=1}^{K}\lambda_{r}s_{r}v_{r}^{T}$$

where **s**_*r *_and **v**_*r *_respectively represent the *r*^*th *^column of **S **and **V**, corresponding to the *r*^*th *^eigenvalue. It can be seen from Equation (3) that the larger the eigenvalue the more it contributes to matrix **G**. Hence, to reduce the dimensionality, one can simply discard the smaller values in the diagonal matrix **Σ **and keep the top *R *eigenvalues. Accordingly, by using the first *R *columns of **S **and **V**, the sizes of the three matrices **S**, **Σ**, **V **are reduced to *M *× *R*, *R *× *R *and *M *× *R*, respectively. Therefore, the number of features is reduced to *R*. Note that **SΣ **gives coordinates of rows of **G **in the space of *R *principle components, and rows of **V**^*T *^are eigenvectors of **G**^*T*^**G**.

Given a new gene vector *G*_*i *_= [*G*_*i*1_,...,*G*_*iM*_] (i.e. the vector for the *i*^*th *^gene), we can project *G*_*i *_into the *R *dimensional subspace as follows:

(4) $${G^{\prime }}_{i}=G_{i}V$$

where ${G^{\prime }}_{i}$ is the new vector with dimensionality *R *(*R *≪ *M*).

### Annotating genes with positive samples

In general, one needs both positive and negative samples to train the classifier, which is subsequently used to make classification. However, there are usually no negative samples in practice for gene function prediction because the available information to us is only about positive samples as described in previous sections. On the other hand, it is inappropriate to use genes currently not annotated with the target function as negative samples because some of them may actually have the function. Furthermore, the number of negative samples generated in this way is much larger than the one of positive samples, which will cause the imbalanced problem and degrade the performance of the classifier [20].

In the literature, some single class learning techniques have been proposed [22-26], e.g. one-class SVMs [21], to distinguish one class of data from the others in the feature space. The one-class SVMs can avoid the imbalanced problem by learning only from the positive set, where it draws a decision boundary to cover most of the positive samples in the feature space. However, there are only a small number of genes annotated in each class here. In other words, there are only a few positive samples available. Therefore, without a negative set, one-class SVMs trained only on a few positive samples tends to overfit easily.

To overcome these problems, we propose a new algorithm, namely Annotating Genes with Positive Samples (AGPS), in this paper. Here, genes with target annotation are denoted as the positive set **P**, genes without target annotation (i.e. annotated with other functions) are denoted as the unlabeled data **Ku**, and unknown genes (i.e. without any annotation) are denoted as **Ug**. In our study, for a specific biological function, the genes without target annotation (**Ku **in this case) are regarded as unlabeled data instead of negative samples because genes are generally annotated with multiple functions and it is inappropriate to simply define those genes not annotated with the target function currently as negative samples. Our goal is to predict the functions of unknown genes based on **P **and **Ku**, where the AGPS algorithm is able to define the negative set automatically in the learning procedure given positive and unlabeled data. The idea behind the AGPS algorithm is to find a subset of negative samples from unlabeled data, where the defined negative set can best recover the positive samples hidden in the unlabeled data. Furthermore, the defined negative set may be a small part of the true negative set but can represent the whole negative set well and avoid the imbalanced problem because the defined negative set can best recover the positive samples from unlabeled data and has a reasonable size. To approach this goal, the positive set is divided into positive training set **P1 **and validation set **P2**, where **P2 **is put into **Ku **to form a new unlabeled data **U **(i.e. **U **= **P2 **+ **Ku**). Although **U **is unlabeled data, the label of **P2 **is known. Therefore, we can select those samples in **U **to best recover **P2 **from **U**. In this procedure, we define a set of samples in **U **as representative negative samples **TN**, with which we get the best prediction results on validation set **P2**. The AGPS algorithm consists of two stages: (1) Learning; (2) Classification. The flowchart of AGPS algorithm is shown in Table 1. In the first stage, one-class SVMs is first utilized to draw an initial decision boundary to cover most of the whole dataset including positive and unlabeled data. The data points not covered by the generated decision boundary are regarded as negative data points because these data points are far from the positive set in the feature space. With the generated initial negative dataset, the two-class SVMs is then employed to expand and refine the negative set from unlabeled data, where each classifier is trained on the positive training set and the negative set generated in the previous iteration, and the trained classifier is subsequently used to classify the remaining unlabeled data. The classifier and negative set generated in each step are recorded. This procedure continues until the stopping criteria are satisfied.

It can be seen that the error in the previous step will affect the current step. To reduce the accumulative error and to avoid the imbalanced problem, the size of the extracted negative samples in the current step is set to |**N**_**cur**_| ≤ *k*|**P**|, where **N**_**cur **_is the predicted negative genes corresponding to the top *n *(*n *≤ *k*|**P**|) smallest decision values by SVMs and *k *is set to 3 in this work so that the false negatives can be reduced to some extent. The negative training set is then set to **N **= [**N**_**cur**_; **N**_**sv**_], where **N**_**sv **_denotes the set of negative support vectors (SVs) and **N**_**cur **_is **N**_**2 **_in Table 1. The idea behind this is that the negative SVs represent well the previous negative training set, and it is not necessary to merge the previous negative set into the current extracted negative set. Thus, the size of negative set is controlled. After obtaining the negative sets and the trained classifiers, one needs to find out the best classifier that can recover the largest number of positive samples lied in the unlabeled data because the classifiers trained above have different discriminative power on the left unlabeled data. The discrimination ability of the trained classifiers is evaluated with *F*1 defined in the next section. Accordingly, the negative set corresponding to the best classifier is returned as the representative negative samples, and the selection of negative samples in this way can help reduce the false negatives to some extent. With the positive and negative samples available, it is straightforward to predict the function of unknown genes in the same way as the conventional two-class SVMs does.

It can be seen from above that there are some similarities between AGPS and the existing methods, i.e. PSoL [23] and SVMC [22]. For AGPS and SVMC, both algorithms use one-class SVMs to construct the initial negative set and two-class SVMs to refine the negative set. On the other hand, for AGPS and PSoL, both algorithms use two-class SVMs to refine the negative set during negative set expansion. However, there are many differences among these three algorithms. The goal of AGPS is to refine a subset of negative samples from unlabeled data, where the defined negative set may be a small part of the true negative set but can represent the whole negative set well and also avoid the imbalanced problem because the defined negative set best recovers the positive samples from unlabeled data and has a reasonable size. With the defined negative set and existing positive set, AGPS employs two-class SVMs to classify unknown genes. On the other hand, the PSoL and SVMC algorithms aim to find possible positive samples from unlabeled data. However, since there are much more negative samples than positive ones in the unlabeled data, the putative positive samples may contain false positives. Furthermore, both PSoL and SVMC do not select the best classifier trained in the learning procedure and the best discriminative negative samples as AGPS does. Therefore, the negative samples generated in PSoL and SVMC may contain more false negatives compared against AGPS. The details of PSoL and SVMC can be found in [23] and [22], respectively.

### Evaluation measures of performance

In this study, the *precision*, *recall *and *F*1 measures are used to evaluate the performance of the classifiers, and defined as follows:

(5) $$precision=\frac{TP}{TP+FP}\times 100\%,$$

(6) $$recall=\frac{TP}{RP}\times 100\%,$$

(7) $$F1=\frac{2*precision*recall}{precision+recall}\times 100\%,$$

where *TP *is the number of genes having function **F **and predicted correctly, *FP *is the number of genes predicted to have function **F **but actually not, and *RP *is the real number of genes that have function **F**.

## Authors' contributions

XZ and LC conceptualized and designed the method. XZ and YW developed the software. LC and KA analyzed and interpreted the data on its biological content. The manuscript was written by XZ and YW, and LC and KA revised it critically. All authors read and approved the final manuscript.

## Supplementary Material

Additional file 1 The functional classes and corresponding genes used in 10-fold cross validation. The names of genes in each class are listed, and these genes have been annotated in MIPS until 2004. Click here for file

Additional file 2 The functional classes and corresponding genes used in test stage. The names of genes in each class are listed, and these genes are not annotated in 2004 but annotated in MIPS in 2006. Click here for file

Additional file 3 The predicted functions of those genes that are not annotated until 2006. These unknown gene are annotated with only the selected 13 functional classes by AGPS. Click here for file
